# Robert Battey, M. D.

**Published:** 1859-09

**Authors:** 


### ROBERT BATTEY, M. D.

Dr. Battey, of Rome, Ga., one of the most promising medical men in the State of Georgia, and a valued contributor to our Journal, will shortly sail for Europe, where he will spend a twelve-month in the pursuit of medical knowledge, to which he is a most assiduous and untiring votary.

He has promised, at our earnest solicitation, to keep up a frequent and regular correspondence with our Journal, which we can assure our readers in advance, will be exceedingly interesting and valuable.

Dr. Battey informed us in an interview we had with him upon the eve of his departure, that he had effected what he regarded as an important modification and improvement in the apparatus for the treatment of Vesico Vaginal Fistula, which he proposed submitting to the eminent surgeons of Europe, by the performance of the operation and a personal application of the proposed improvement in the apparatus, should an opportunity offer.

